# Fractures of the Shaft of the Ulna

**Published:** 1858-08

**Authors:** Frank Hastings Hamilton

**Affiliations:** Buffalo


### BUFFALO MEDICAL JOURNAL

AND

### MONTHLY REVIEW.

**VOL. 14.** AUGUST, 1858. **NO. 3.**

### ORIGINAL COMMUNICATIONS.

ART. I.—*Fractures of the Shaft of the Ulna.* By Frank Hastings Hamilton, M. D., Buffalo.

*Causes.* The shaft of the ulna is generally broken by a direct blow. I have never seen an exception to this rule; but Voisin has related in the Gazette Médicale for 1833, a single example in which it was said to have been broken by a fall upon the palm of the hand. Malgaigne thinks it is most often broken when one seeks to ward off a blow with the arm; but it has happened most often to me to see it broken by a fall upon the side of the arm.

*Point of Fracture, Direction of Displacement, &c.* In an analysis of twenty-two cases, I find the shaft has been broken seven times in its upper third, seven times in its middle third, and eight times in its lower third. All portions seem, therefore, to be about equally liable to fracture. I think, also, the fracture has generally been oblique.

Contrary to what has been observed by other writers, I have noticed that no law prevailed as to the direction in which the fragments have become displaced; the broken ends being found directed forwards, backwards, inwards or outwards, according to the direction of the blow which has occasioned the fracture; and this is in accordance with the general rule in other fractures occasioned by direct blows. No doubt, however, other things being equal, the tendency of the lower fragment would be towards the inter-osseous space, in consequence of the action of the pronator quadratus in this direction, and if the fracture is above the middle, the pronator radii teres also will increase this tendency; while the upper fragment, owing to its broad and firm articulation at the elbow-joint, can only be displaced forwards or backwards, at least to any great extent.

*Complications.* In no fracture of a long bone have I found serious complications so frequent as in fractures of the shaft of the ulna. Three have been compound; seven complicated with a forward dislocation of the head of the radius; one with a partial dislocation of the lower end of the radius backwards, and one with a dislocation of both radius and ulna backwards at the elbow-joint. It will be seen, therefore, that twelve, or more than one-half of the whole number, have been seriously complicated.

*Symptoms.* Occasionally this fracture is found to exist without sensible displacement. In such cases the diagnosis is sometimes difficult, and can only be determined by the crepitus and mobility. If, however, the ulna is firmly seized above and below the point which has suffered contusion, and pressed in opposite directions, these signs will generally be sufficiently manifest, and will render the diagnosis certain.

But in cases where there is considerable displacement, the inner surface of the bone is so superficial as to enable us to detect its deviations with the eye alone, or, when swelling has already occurred, by the fingers carried firmly and slowly along this margin.

If the head of the radius is dislocated also, the displacement of the broken ends of the ulna must always be considerable, and the consequent deformity palpable. I have known one instance, however, in which a surgeon living in the neighboring Province of Upper Canada, recognized and reduced a dislocation of the radius and ulna backwards, but did not detect a fracture of the ulna two inches above its lower end. Six months after, in the month of March, 1856, he called upon me with a marked deformity near the wrist, occasioned by the backward projection of the broken ulna, and with a complete loss of the power of supination. It will not surprise us that this fracture was overlooked when we learn that the man had fallen fifty-five feet.

*Prognosis.* In simple fractures the prognosis is generally favorable, since no overlapping can occur, and the lateral displacements are not usually sufficient to produce a marked deformity, or to interfere materially with the functions of the arm; yet it is not unfrequent to find the fragments inclining slightly forwards or backwards, inwards or outwards. If the fragments fall towards the radius, I have noticed in three or four instances a slight projection of the lower end or styloid process of the ulna to the ulnar side; but not interfering in any degree with the motions of the wrist-joint.

I have seen the radius left unreduced three times after a fracture of the ulna, and in each example the forearm is shortened. A boy, æt. 17, was struck by a locomotive, and severely injured in various parts of his body, June 5, 1855. I saw him with two very intelligent country practitioners, a few hours after the accident. The whole left arm was then greatly swollen. Crepitus was distinct, and we easily recognized the fracture of the ulna about three inches below its upper end, with which an open wound was in direct communication. We suspected also, a dislocation of the head of the radius forwards, but as we could not make ourselves certain, and finding that the arm was in such a condition as to preclude any farther manipulation without greatly diminishing the chance of saving the limb, we made no attempt at reduction, but laid the arm upon a pillow and directed cool water lotions.

At no subsequent period, in the opinion of the medical gentleman who was left in charge, did a favorable opportunity occur to reduce the radius; and at the end of two months I found the ulna united, with the fragments bent forwards and outwards towards the radius, while the head of the radius lay in front of the humerus. The forearm was shortened three-quarters of an inch. He could flex his arm freely to a right angle and a little beyond; and he could straighten it perfectly. Hand slightly proned, with partial loss of supination. Whole arm nearly as strong and as useful as before the accident. Above the olecranon process, on the back of the humerus, I observed a remarkable fullness occasioned by the shortening of the triceps muscle.

The second case occurred in the person of a man æt. 26, residing about twenty miles from town, and was occasioned by the kick of a horse. This was also a compound fracture. It does not appear that his surgeon discovered the dislocation of the radius, but supposed that it was a fracture of both bones. On the ninth day the patient became dissatisfied and dismissed his surgeon, but employed no other.

Oct. 1, 1849, eleven weeks after the accident, he called upon me at Buffalo. I found the ulna united with a manifest displacement, but I could not discover that there had been any fracture of the radius. The head of the radius was in front of the external condyle, and a depression existed where it formerly articulated. When the arm was flexed, the head did not strike the humerus so as to arrest the flexion, but it glided upwards and outwards along the inclined base of the external condyle. He had already begun to use his arm considerably in labor. The forearm was shortened one inch.

The third example was in the person of John Lewis, of Pa., æt. 25, who told me, in Sept. 1851, that his left ulna had been broken two years before, and at several points. He was attended by two surgeons living at Montrose, Pa.

I found the ulna much bent forwards a little below its middle, the head of the radius displaced forwards, and the forearm shortened one inch.

Three times I have noticed after the lapse of several years that the forearm could not be perfectly supined; but pronation was never permanently impaired. I think, also, that the motions of flexion and extension have always, except where the radius has remained dislocated, been completely restored soon after the splints were removed; and even in these latter cases, it is only extreme flexion which has been hindered.

*Treatment.* In simple fracture we must look carefully to the lateral deviation of the fragments, and if they are found to be salient forwards or backwards, pressure made directly upon or near their extremities, restores them to place, but it often requires considerable force to accomplish this. A gentleman fell and broke the right ulna near its middle. He came immediately to me, and I found the fragments displaced backwards. Pressing strongly with my fingers, they sprung forwards with a distinct crepitus, and I thought they were now in exact line. A broad and well padded splint was applied to the forearm, and I took especial pains with compresses nicely adjusted, from day to day, to keep every thing in place. The arm was placed in a sling. Eight months after the accident the gentleman died of cholera, and I was permitted to dissect the arm. I found the fragments well united but with a very palpable projection of the fragments backwards, in the direction which I first found them.

If the displacement is in the direction of the radius, it is more difficult to overcome, but its necessity is much more urgent, since if the fragments fall completely against the radius, a bony union may take place, occasioning a complete loss of the power of pronation and of supination.

While moderate extension is being made, and the head is firmly supined, the fingers of the surgeon should be pressed firmly, and in spite, sometimes, of the complaints of the patient, between the radius and ulna, and the fragments of the broken ulna fairly pulled out from the radius.

The forearm may now be laid in the usual position against the front of the chest, midway between supination and pronation, and the same splints applied and in the same manner which we shall hereafter describe for fractures of the shaft of both bones.

We ought, however, especially to bear in mind the danger of drawing the fragments against the ulna, by allowing the sling or the bandages to rest against the middle of the ulnar side of the bone. To prevent this, the sling ought to support the arm by passing only under the hand and wrist, or the forearm may be laid in a firm gutter which will touch the forearm only at the elbow and wrist, or it may be laid upon its back as suggested and practised by Flewry, who, according to Malgaigne, had a case which had been treated in the position of semi-pronation, and which remained not only displaced but refused to unite; but when the arm was supined the fragments came at once into contact and bony union speedily took place. This position may be adopted whenever it is found to be practicable; but the position of demi-pronation is generally much more comfortable to the patient, at least when the forearm is laid across the chest, and very few patients will submit to a position of complete supination.

In fractures accompanied with dislocation of the head of the radius forwards or backwards, nothing should prevent the immediate reduction of the dislocation but a demonstration of its impossibility, or a condition of the limb which would render manipulation hazardous. It can be reduced generally by pushing forcibly upon the head of the bone in the direction of the socket, while the arm is moderately flexed so as to relax the biceps, and while extension is being made at the forearm by an assistant. In making the counter-extension care should be taken to seize the lower end of the humerus by the condyles, rather than by its anterior aspect, by which precaution we shall avoid pressing upon and rendering tense the tendon of the biceps.

July 29, 1845, a lad, æt. 9, fell from his bed, breaking the ulna near its middle and dislocating the head of the radius forwards. Dr. Austin Flint was called on the following morning, and at his request I was invited to see the patient with him. We found the ulna broken obliquely near its middle, and the head of the radius dislocated forwards. While Dr. Flint seized the elbow in front of the condyles, I made extension from the hand, the forearm being slightly flexed upon the arm, and at the same moment I pushed forcibly the head of the radius back to its socket.

We then dressed the arm with Rose's angular splints, constructed with a joint opposite the elbow. This was laid upon the palmer surface, and the whole was nicely padded, especially in front of the head of the radius. In two weeks pasteboard was substituted for the angular splint. At the end of six weeks I was permitted to examine the arm and found the head of the radius perfectly in place, but the points of fracture slightly salient. All of the motions of the arm were completely restored.

June 2, 1845, C. C., æt. 9, fell upon his arm breaking the ulna obliquely near its middle, and dislocating the head of the radius forwards. Dr. J. P. White being called, requested me to visit the patient also with him. We found one of the broken fragments protruding through the skin, on the inside of the arm.

With great ease, and by simply pressing with considerable force upon the head of the radius, it was made to slide into its socket. The case was left in charge of Dr. White.

Five weeks after I found all of the motions of the forearm completely restored, except that he could not extend it perfectly. The head of the radius was also a little more prominent in front than in the opposite arm.

Four or five years afterwards, the projection of the head of the radius had disappeared and the functions of the arm were perfect.

The following example of compound and comminuted fracture of the ulna will illustrate how much may be accomplished by conservative surgery:

A German lad, æt. 10, was run over by a railroad car, Sept. 4, 1857. Drs. C. C. F.Gay and Austin Flint, Jr., were summoned immediately; but the limb presented such a discouraging appearance as induced them to send for me also.

We found the ulna very much broken near its lower end, and about two inches of it entirely gone. The radius was sound. The skin and muscles were extensively lacerated and torn off in shreds.

After a careful examination, finding that the radial and ulnar arteries continued to pulsate, upon consultation together, we agreed to attempt to save the limb. It was accordingly laid upon a board covered with a soft and nicely adjusted cushion; such vessels as were bleeding were tied; the skin was loosely stitched together, and the whole covered with a cotton cloth smeared with simple cerate. Cool water dressings were directed, and the boy was left in charge of Drs. Gay and Flint. The skin subsequently sloughed extensively, and also more or less of the muscular tissue; but on the 1st of May, 1858, about eight months from the time of the accident, it had nearly or quite closed over, and although his arm was very much deformed and maimed, it was still very useful, indeed to one who must earn his living by his hands alone, its value is beyond estimate.

